# Enhancing Medium-Chain Fatty Acid Delivery Through Bigel Technology

**DOI:** 10.3390/gels10110738

**Published:** 2024-11-14

**Authors:** Manuela Machado, Eduardo M. Costa, Sara Silva, Sérgio C. Sousa, Ana Maria Gomes, Manuela Pintado

**Affiliations:** CBQF-Centro de Biotecnologia e Química Fina—Laboratório Associado, Escola Superior de Biotecnologia, Universidade Católica Portuguesa, Rua Diogo Botelho 1327, 4169-005 Porto, Portugal; emcosta@ucp.pt (E.M.C.); snsilva@ucp.pt (S.S.); sdsousa@ucp.pt (S.C.S.); amgomes@ucp.pt (A.M.G.); mpintado@ucp.pt (M.P.)

**Keywords:** release properties, medium-chain fatty acids, rheology, permeability, bigel technology, nutraceutical applications

## Abstract

This study presents the development and characterization of medium-chain fatty acid (MCFA)-loaded bigels, using coconut oil as the MCFA source. The bigels exhibited high oil binding capacity, ranging from 87% to 98%, effectively retaining MCFAs within the matrix, with lauric acid (C12) being the main component detected within the bigels at 178.32 ± 0.10 mg/g. Physicochemical analysis, including FTIR and scanning electron microscopy, confirmed stable fatty acid incorporation and a cohesive, smooth structure. The FTIR spectra displayed O-H and C=O stretching vibrations, indicating hydrogen bonding within the matrix, while the SEM images showed uniform lipid droplet distribution with stable phase separation. Thermal stability tests showed that the bigels were stable for 5 days at 50 °C, with oil retention and structural integrity unchanged. Rheological testing indicated a solid-like behavior, with a high elastic modulus (G′) that consistently exceeded the viscous modulus (G″), which is indicative of a strong internal structure. In simulated gastrointestinal digestion, the bigels achieved significantly higher MCFA retention than the pure oil, particularly in the gastric phase, with recovery percentages of 38.1% for the bigels and 1.7% for the oil (*p* < 0.05), suggesting enhanced bioavailability. Cell-based cytotoxicity assays showed low cytotoxicity, and permeability testing in a co-culture Caco-2/HT29-MTX model revealed a controlled, gradual MCFA release, with approximately 10% reaching the basolateral side over 6 h. These findings highlight MCFA-loaded bigels as a promising platform for nutraceutical applications; they provided stability, safety, and controlled MCFA release, with significant potential for functional foods aimed at enhancing fatty acid bioavailability.

## 1. Introduction

The bioavailability of nutrients and bioactive compounds is a fundamental factor in determining their effectiveness in health promotion and disease prevention [1]. Among bioactive compounds, medium-chain fatty acids (MCFAs) are gaining significant attention in nutraceutical applications due to their unique metabolic properties and associated health benefits, including immunomodulatory effects, cholesterol regulation, and potential roles in weight management [2]. However, the effective delivery and absorption of MCFAs are still limited as, due to their lipophilic nature, these compounds present poor water solubility and a tendency to crystallize, which contribute to their reduced bioavailability [3,4].

To address these challenges, encapsulation techniques have emerged as effective strategies for enhancing the stability and bioavailability of lipophilic compounds like MCFAs. Among these approaches, bigels—semi-solid systems formed by combining hydrogel and oleogel phases—are proving to be highly effective as they possess several advantages: the combination of hydrogel and oleogel phases provides enhanced structural stability, while their biphasic nature enables the co-delivery of both lipophilic and hydrophilic bioactive compounds, broadening their potential applications [5,6]. Moreover, bigel formulations can be customized for specific release profiles by adjusting the hydrogel/oleogel ratio, allowing precise control over the timing and extent of compound release [7,8,9]. Examples of this potential can be found in recent studies, with bigels being capable of protecting and enhancing the bioavailability of bioactive fatty acids and other lipophilic compounds, such as vitamin E, lycopene, and β-carotene, by stabilizing these compounds and improving their oxidative stability, thus maximizing their biological potential [10,11,12,13].

Given the information presented above, the rationale of this work was that encapsulating MCFAs within a bigel matrix would enhance their absorption and bioavailability, offering a delivery system that could overcome the solubility and crystallization challenges associated with MCFAs for potential nutraceutical applications. To do so, a detailed analysis of the physicochemical properties of bigels, as well as their interaction with biological systems, was performed by examining factors such as oil binding capacity, thermal stability, and gastrointestinal protection, thus demonstrating the advantages of bigels in delivering MCFAs effectively and safely and supporting their development as advanced delivery platforms in functional foods and nutraceuticals.

## 2. Results and Discussion

### 2.1. Bigel Physicochemical Characterization

Medium-chain fatty acid-loaded bigels were successfully prepared using coconut oil as a source of MCFAs, with a 1:1 ratio of hydrogel to oleogel phases [11]. The bigels demonstrated high oil binding capacity with values ranging between 87 and 98%, indicating effective oil retention within the gel structure. The oil binding capacity data were consistent with those previously reported for coconut oil bigels (ranging between 92 and 98%) and for bigels with other oils, such as walnut oil (varied between 88 and 96%) [11,14]. Lauric acid (C12), which was the MCFA predominantly identified in coconut oil, was also the main component quantified in the bigels; this was consistent with prior studies on coconut oil encapsulation within bigels [11].

As shown in Table 1, the bigel fatty acid profile reflects a broad MCFA retention, though concentrations of individual fatty acids are notably lower than in pure coconut oil. Statistically significant differences were observed for several fatty acids, namely C6 (caproic acid), C8 (caprylic acid), C10 (capric acid), and particularly C12 (lauric acid), of which the bigels contained 178.32 ± 0.10 mg/g compared to the 412.86 ± 7.85 mg/g present in the oil (*p* < 0.001). This reduction can be explained with the encapsulation effects of the bigel matrix, which disperses fatty acids within the hydrogel–oleogel structure, lowering their overall concentration while maintaining the fatty acid profile. The cumulative MCFA content (∑MCFA) in the bigels was 231.25 ± 0.21 mg/g, which was significantly lower than that in the coconut oil (893.45 ± 12.0 mg/g, *p* < 0.001); yet, it still accounted for a substantial proportion of the total fatty acids in the bigel (∑Fatty acids 401.81 ± 0.43 mg/g compared to 562.39 ± 8.96 mg/g in oil, *p* < 0.001). The lower concentration of fatty acids in the bigels is probably due to their distribution across the gel matrix, which stabilizes them and reduces their direct availability. The fatty acids C18:1 c11 (cis-vaccenic acid) and C18:3 (linolenic acid) showed no significant differences (*p* > 0.05) between the bigels and oil, indicating that these components may integrate similarly within both structures.

These findings reinforce the stability and retention of MCFAs within bigel matrices, showcasing their potential as controlled-release systems for MCFAs in foodstuffs and nutraceutical applications. The bigel formulation’s ability to encapsulate MCFAs efficiently, while maintaining a significant fatty acid profile, suggests a promising approach for enhancing the delivery and bioavailability of coconut oil’s beneficial fatty acids [11].

**Figure 1:**
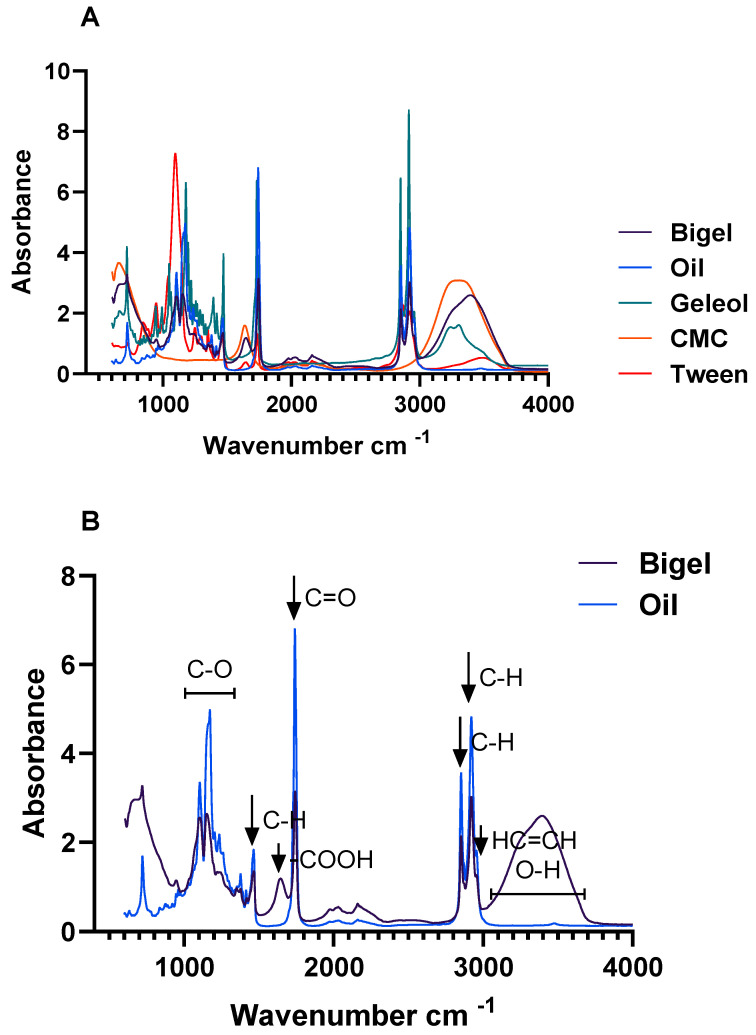
(**A**): FTIR spectra of bigels and their components (coconut oil, geleol, CMC, and tween 80). (**B**): Identification of the major functional groups in oil and bigel FTIR spectra.

In terms of possible interactions between hydrogels within the bigel system, the FTIR spectra (Figure 1) showed a broad band at 3000–3700 cm^−1^, which could be related to the O-H stretching vibrations participating in the hydrogen bonds within carboxymethyl cellulose (CMC) and the acyl groups present in the oleogel fraction [12]. The absorption peaks related to the C-H and HC=CH bonds (2920 and 2958 cm^−1^) represented the stretching vibration of the acyl chains of fatty acids [12]. The presence of these bonds shows that the fatty acids were well incorporated into the bigel structure. The absorption peak at 1474 cm^−1^ corresponded to the stretching vibration of the C-H bonds in the saturated fatty acids (the main fatty acids present in coconut oil). The characteristic absorption peak related to the C=O stretching vibration was observed at 1750 cm^−1^ [15,16]. The absorption peak at 1660 cm^−1^ represented the stretching vibration of the carboxyl group of CMC, indicating that it remained integrated within the bigel structure and suggesting the presence of hydrogen bonds within the oleogel fraction. Finally, the bands between 1400 cm^−1^ and 1000 cm^−1^ can be associated with the stretching vibration of C-O in the ester group [15,16].

**Figure 2:**
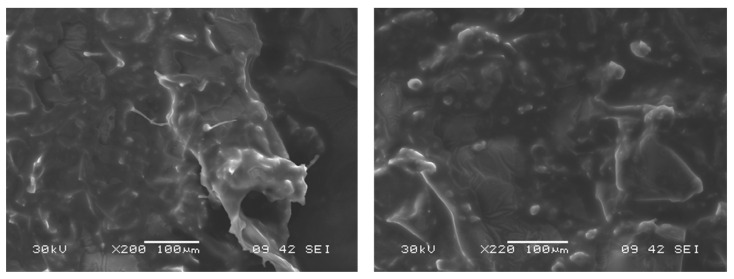
The microstructure of bigels using SEM technology.

Concerning the bigel’s microstructure (Figure 2), SEM images provided a close-up view of its microstructure at a magnification of 200× and scale of 100 μm. The images highlighted the distribution of lipid droplets within the gel network, which is characteristic of the 50% oleogel and 50% hydrogel composition. The smoothness observed in the structure can be attributed to the presence of monoglycerides as oleogelators, which contributed to the uniform and cohesive texture throughout the bigel structure [14]. Additionally, the images also showed various spherical and irregularly shaped droplets distributed within the hydrogel matrix, indicating a stable phase separation essential for maintaining a cohesive bigel network. This distinct phase separation not only supports the bigel structural integrity but also likely enhances its mechanical properties and stability, making it well suited for various applications in food and nutraceutical delivery systems.

The organized microstructure observed in these SEM images underscores the bigels’ potential to provide a controlled release and improve the bioavailability of encapsulated compounds due to their stable, multi-phase matrix [17].

### 2.2. Bigel Thermal Stability

**Figure 3:**
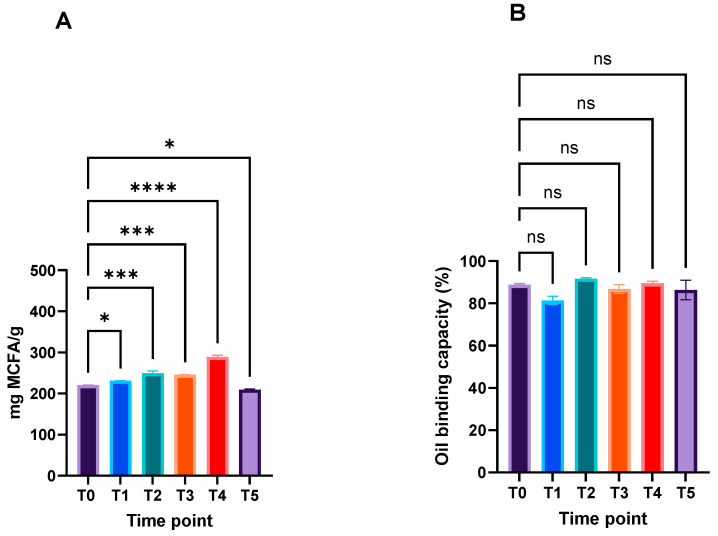
Impact of temperature on bigel’s MCFA amount (**A**) and oil binding capacity (**B**) during the 5 days of storage at 50 °C. ns means no significant differences (*p* > 0.05), * means significant differences (*p* < 0.05), and *** means significant differences (*p* < 0.001) and **** means significant differences (*p* < 0.0001).

According to the literature, coconut oil exhibits a low oxidation rate [18,19]. Despite this, the MCFA fraction in the bigels after 5 days at 50 °C showed statistically significant differences (*p* < 0.05), when compared to the freshly prepared bigel (T0) (Figure 3A). The amount of MCFAs varied between 220 ± 0.77 mg/mL and 209.48 ± 1.79 mg/mL.

In terms of its oil binding capacity, the results obtained showed no significant differences (*p* > 0.05) during the thermal stability assay (Figure 3B). This indicates that the gel structure was not affected by the temperature tested.

**Figure 4:**
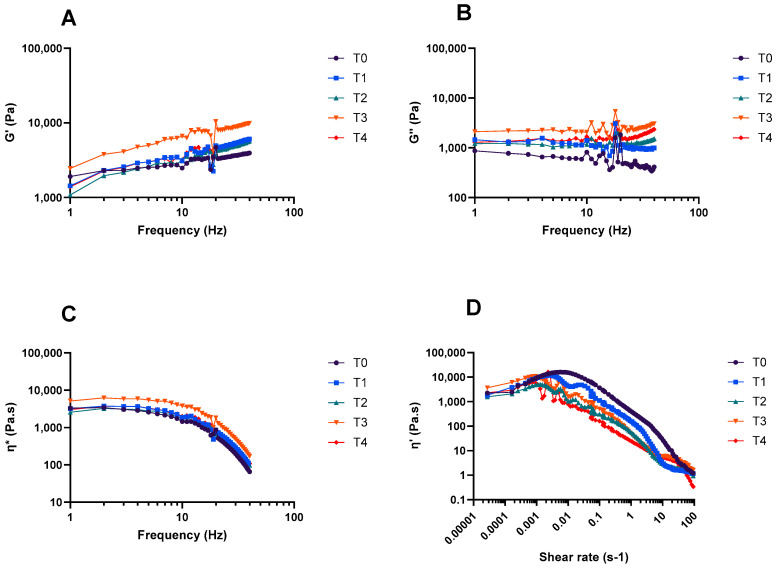
Rheological properties of bigels during thermal stability (T0—day 0, T1—day 1, T2—day 2, T3—day 3, T4—day 5); (**A**) elastic modulus G′; (**B**) viscous modulus G″; (**C**) complex viscosity η*; (**D**) instantaneous viscosity η′.

The bigel viscoelastic properties were evaluated by performing the oscillatory test, to estimate the elastic modulus (G′) and viscous modulus (G″) and complex viscosity. As can be seen in Figure 4, the bigels presented a solid-like behavior (G′ was higher than G″ within the linear viscoelastic region) [20]. The lack of an intersection between G′ and G″ (no point where G′ equals G″) across the studied frequencies suggests that the bigel did not undergo a gel-to-sol transition. This observation may imply the presence of stronger internal forces within the bigel, contributing to its solid-like behavior [21,22,23]. A similar occurrence was previously reported for bigels loaded with different vegetable oils [11]. Slight differences in the rheological properties were observed during the thermal stability assessment. The complex viscosity slightly increased due to the increase in G′ and viscous G″. The increase in G′ and G″ could be due to the interaction between the oil fraction and the carboxymethyl cellulose hydrogel [23]. These interactions occur when the polymer molecules receive adequate energy to overcome intermolecular repulsion [23]. Regarding the viscometry assay (Figure 4D), the results show that an increase in the shear rate decreases the apparent viscosity (η′) of the MCFA-loaded bigel. Previous works using coconut and fish oil bigels showed a similar trend: the increase in shear rate led to a decrease in the bigel’s viscosity [11,24].

### 2.3. Impact of Gastrointestinal Tract

**Figure 5:**
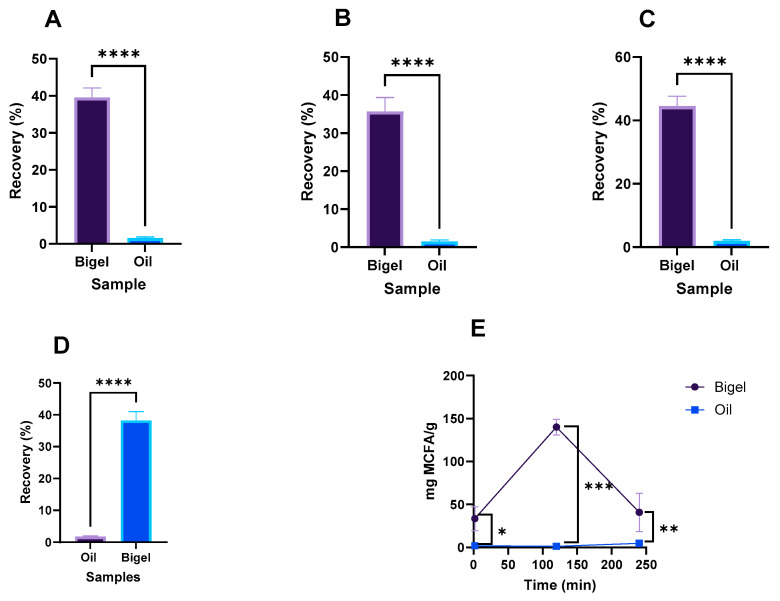
Recovery percentages after gastrointestinal tract: saturated (**A**), monounsaturated (**B**), polyunsaturated (**C**), and medium-chain fatty acids (**D**). The release profile of MCFAs during gastrointestinal tract (**E**). * means significant differences at (*p* < 0.05), ** means significant differences (*p* < 0.01), *** means significant differences (*p* < 0.001) and **** means significant differences (*p* < 0.0001).

The pure oil and bigels were subjected to gastrointestinal digestion to understand how it affected them and whether bigels have a protective effect that could enhance their subsequent bioavailability. As can be seen in Figure 5, the gastrointestinal tract significantly (*p* < 0.05) affected the fatty acid content, with recovery percentages lower than 50% in the case of the bigel and of less than 10% in the case of the pure oil. The recovery percentages of saturated fatty acids (Figure 5A) ranged between 36.6 and 41.6% for the bigel and between 1.4 and 1.6% for the oil. In the case of monounsaturated fatty acids (Figure 5B), the values ranged between 36.1 and 43.7 and 1.8 and 2.2 for the bigel and oil, respectively. For the polyunsaturated fatty acids (Figure 5C), the recovery rate was between 39.0 and 43.9 and 1.1 and 2.04 for the bigel and oil, respectively. These results are in line with those previously reported for coconut oil bigels [11]. Focusing on the MCFAs, the recovery percentage ranged between 38.14 ± 2.74 for the bigel and 1.73 ± 0.28 for the pure oil (Figure 5D). The release of MCFAs in the bigel occurred mainly in the gastric phase (Figure 5E). According to some researchers, this can be related to the low pH values and the swelling properties of bigel [12,21,25].

### 2.4. Cell-Based Assays

#### 2.4.1. Cytotoxicity

**Figure 6:**
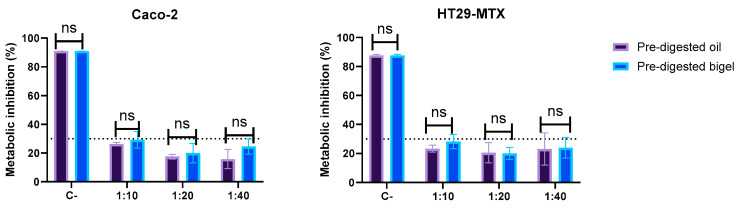
Effect of digested oil and bigel upon Caco-2 and HT29-MTX metabolism. ns means no significant differences (*p* > 0.05). The dotted line represents the 30% cytotoxicity limit, as defined by the ISO 10993-5 [26].

As can be seen in Figure 6, the bigel and oil digested fractions had a slight cytotoxic effect at the first tested concentration (corresponding to a concentration of 6 mg/g of MCFAs). At the remaining concentrations, no deleterious effects were observed in either of the cell lines. For this reason, for the subsequent analysis, the 1:20 dilution was used (which represents 3 mg/ mL and 2.9 mg/mL of MCFAs for the bigel and oil, respectively).

This low cytotoxicity across most concentrations underscores the bigels’ potential for human applications, particularly in nutraceuticals, where safety and biocompatibility are crucial. Ensuring minimal cytotoxic effects supports the feasibility of bigels as a delivery system, allowing the controlled release of bioactive fatty acids with minimal risk of adverse cellular reactions and enhancing their suitability for functional food and health applications.

#### 2.4.2. MCFAs Bioavailability 

**Figure 7:**
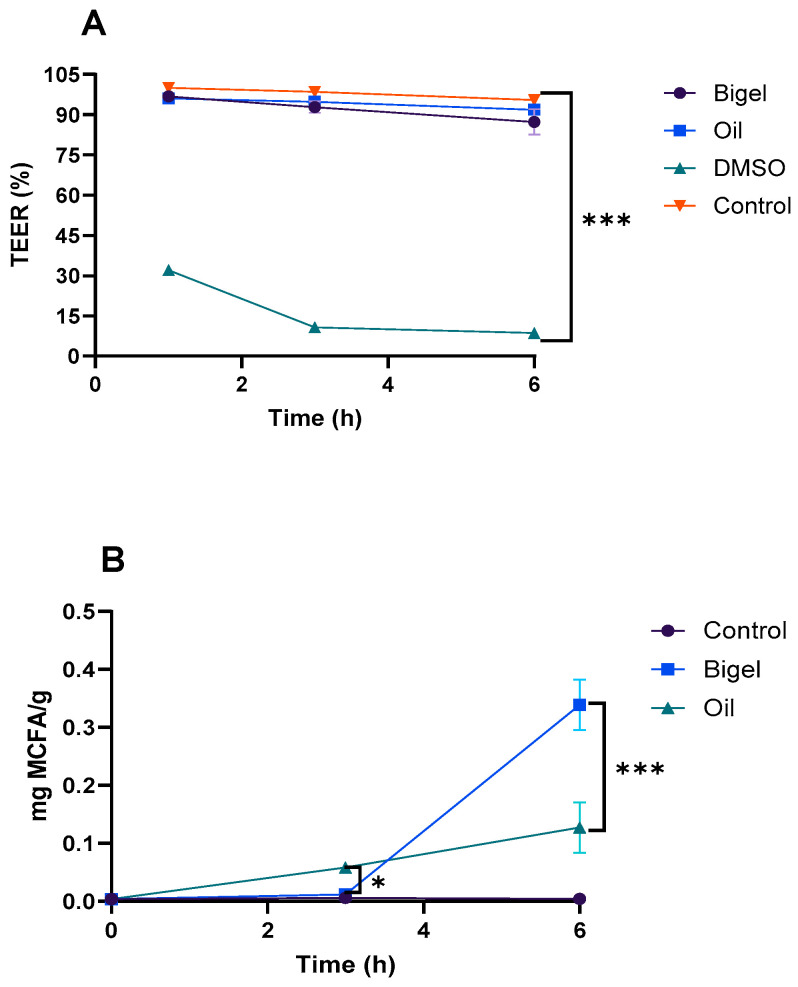
(**A**): Membrane stability as TEER (%) and (**B**): MCFA permeability over 6 h. ns means no significant differences, * means significant differences (*p* < 0.05) and *** means significant differences (*p* < 0.001).

Following the cytotoxicity assessment, a permeability assay was performed to determine whether digested bigel formulations enhanced MCFA bioavailability in comparison to digested oil. Transepithelial electrical resistance (TEER) measurements revealed a decrease in TEER (%) values in the Caco-2/HT29-MTX co-cultures upon exposure to the digested samples (Figure 7A). This drop in TEER aligns with other studies showing similar reductions in electrical resistance in the Caco-2 monolayers and co-cultures exposed to fatty acids and lipid-rich media and may indicate a temporary reduction in membrane integrity, likely due to the hydrophobic nature of the samples [27,28]. Free fatty acids can disrupt cell membrane lipid bilayers, promoting paracellular permeability and increased fluidity, which is associated with greater absorption potential for bioactive compounds [28,29,30,31]. The MCFA permeability results indicated that only a small percentage of MCFAs (approximately 10% for bigels and 6% for oil) reached the basolateral side, with peak absorption observed at 6 h. This gradual transfer likely reflects cellular mechanisms, such as re-esterification and storage, within the intracellular lipid pools, which limited direct transport across the cell layer [32,33]. These findings suggest that bigels may enable a controlled and sustained release of MCFAs, highlighting their potential as targeted delivery systems for nutraceutical applications aimed at optimizing fatty acid absorption.

## 3. Conclusions

This study presents novel MCFA-loaded bigels using coconut oil, developed with a balanced 1:1 ratio of hydrogel to oleogel phases. These bigels demonstrated a high oil binding capacity (87–98%), effectively encapsulating MCFAs, particularly lauric acid, which was retained as the primary component within the gel matrix. The structure analysis showed strong O-H and C=O bonds that contribute to a cohesive gel matrix, and the SEM images showed a uniform distribution of lipid droplets, with monoglycerides creating a smooth texture and stable phase separation. The bigels maintained oil retention and mechanical characteristics under thermal stress and presented a solid-like behavior; both of these characteristics are suitable for extended storage and functional applications. Under GI tract conditions, the bigels improved MCFA retention, enhancing its bioavailability, and the digested fractions revealed low cytotoxicity across most concentrations, while the permeability testing showed that bigels deliver MCFAs gradually, without significant impact on cell membrane integrity. These findings underscore the potential of these MCFA-loaded bigels as effective, stable, and safe delivery systems for bioactive fatty acids, offering controlled release, enhanced bioavailability, and promising applications in functional foods and nutraceuticals.

## 4. Materials and Methods

### 4.1. Bigels Preparation and Oil Binding Capacity Evaluation

**Figure 8:**
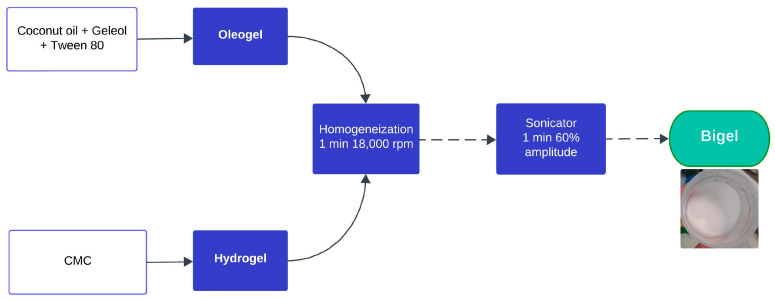
Schematic representation of bigel production.

Coconut oil bigels were prepared following the method of Machado et al., 2023 [11], as shown in Figure 8. In summary, geleol was melted at 60 °C and mixed with pre-heated Tween 80 in a 1:1 (*v*/*v*) ratio. Pre-heated coconut oil (5 g) was then incorporated with continuous stirring, followed by the addition of a pre-warmed hydrogel fraction containing carboxymethylcellulose (2% *w*/*v*). The mixture was homogenized for 1 min at 18,000 rpm using an Ultra-Turrax (IKA T 25 digital, Janke and Kunkel IKA-Labortechnik, Staufen, Germany) and subsequently sonicated with a Sonics Vibra-Cell™ VCX 130 at 60% amplitude for 1 min.

After cooling, the oil binding capacity was assessed by centrifugation, as described by Li et al., 2024 with slight modifications [14]. Briefly, a controlled amount of bigel (1 g) was placed into pre-weighed microtubes (*m*1), centrifuged at 4 °C and 15,000 rpm for 20 min. Excess oil was decanted, and the microtube with the residual gel was weighed (*m*2). The oil binding capacity was calculated using the following formula:

$$OBC\;\%=\left[1-\frac{\left(m1-m2\right)}{m1}\;\right]\times 100$$

### 4.2. Fatty Acid Profile

The fatty acid profile of the bigels was assessed post-transesterification, following the method of Machado et al., 2023 [11]. Analysis was conducted using an Agilent 8860 GC system (Agilent, Santa Clara, CA, USA) with a flame ionization detector and a BPX70 capillary column (60 m × 0.25 mm × 0.25 µm, SGE Europe, Paris, France). The injector (split ratio 25:1) and FID temperatures were set at 250 °C and 275 °C, respectively, with hydrogen as the carrier gas at a flow rate of 1 mL/min and pressure of 20.5 psi. The oven temperature program ranged from 60 °C to 225 °C. The fatty acids were identified using Supelco 37 (Sigma, St. Louis, MO, USA), and each sample was analyzed in triplicate.

### 4.3. ATR-FTIR Spectra

FTIR analysis was performed with a Spectrum 100 FTIR spectrometer (PerkinElmer, Waltham, MA, USA), utilizing the attenuated total reflectance (ATR) technique. Spectral data were gathered across a range from 4000 to 500 cm^−1^ at a 4 cm^−1^ resolution, averaging 32 scans for each sample. Background correction was applied to the acquired spectra.

### 4.4. Microstructure Observation by Scanning Electron Microscopy

The network structure of the bigels was examined using scanning electron microscopy with a JSM-5600LV microscope (JEOL, Tokyo, Japan). The samples were mounted onto double-sided adhesive carbon tape (NEM tape, Nisshin, Japan) and coated with a gold/palladium layer using a sputter coater (Polaron, Bad Schwalbach, Germany). The SEM was operated in high-vacuum mode with a spot size range of 16–30 and an accelerating voltage between 18 and 30 kV.

### 4.5. Thermal Stability

Newly prepared coconut oil-loaded bigels were placed in amber vials and incubated at 50 °C for five days in darkness. Changes in fatty acid profile and oil binding capacity were assessed following the procedures outlined in Section 4.1 and Section 4.2. The effects on the rheological properties were evaluated according to the method in the subsequent section.

### 4.6. Rheological Measurements

Rheological analysis of the bigels stored at 50 °C was carried out using a Gemini Advanced Rheometer (Bohlin Instruments, Cranbury, NJ, USA) equipped with a Peltier temperature control unit and a stainless steel cone-and-plate probe (40 mm diameter, 4° angle). The samples were placed on the measurement plate, and tests were performed with a 1000 µm gap. The elastic modulus (G′), viscous modulus (G″), and complex viscosity (η*) were measured across a frequency range from 0.1 to 100 Hz, using a 0.1% strain, verified to be within the linear viscoelastic region (LVER). Additional viscometry measurements (shear rate ranging from 0.001 to 100 s^−1^) were conducted using a 150 µm gap to analyze the shear behavior of the bigel samples. Each sample was tested at 25 °C in triplicate.

### 4.7. In Vitro Gastrointestinal Tract Simulation

An in vitro gastrointestinal tract simulation was conducted based on the INFOGEST protocol [34]; it was performed in triplicate with 5 g samples of bigels or pure edible oils. The effect of gastrointestinal digestion on fatty acid release was assessed as outlined in Section 2.2, using 0.5 mL of digested material from both the bigel and pure oil samples. The results were then compared to those of the initial, undigested samples.

### 4.8. MCFAs Bioavailability Evaluation

#### 4.8.1. Cell Line Culture Conditions

Human Caucasian colon carcinoma epithelial cells (Caco-2 ECACC 86010202) and HT29-MTX E12 (ECACC 12040401) were acquired from the European Collection of Authenticated Cell Cultures. The cells were cultured using Dulbecco’s Modified Eagle’s Medium (DMEM; Gibco, Thermo Scientific, Waltham, MA, USA) supplemented with 10% (*v*/*v*) fetal bovine serum from Biowest (Nuaillé, France) and 1% (*v*/*v*) of penicillin–streptomycin–fungizone (Lonza, Basel, Switzerland and 1% (*v*/*v*) non-essential amino acids (Gibco, Thermo Scientific, MA, USA). The cell lines were incubated at 37 °C under a humidified atmosphere comprising 5% CO_2_ and 95% air.

#### 4.8.2. Cytotoxicity Assessment

The cytotoxicity assay followed the procedure outlined by Machado et al., 2022 [35]. In brief, the cells were seeded at a density of 1.0 × 10^4^ cells per well in 96-well tissue culture plates. After a 24 h incubation, the medium was replaced with either digested bigels or digested oil, while plain media served as a positive control and media containing DMSO as a negative control. Following 24 h of exposure, 100 µL of MTT solution (0.5 mg/mL) was added to each well, and the plates were incubated for 2 h. The supernatant was then carefully removed, and 100 µL of DMSO was added to dissolve the formazan crystals. Absorbance was measured at 570 nm using a Synergy H1 microplate reader (Biotek Instruments, Winooski, VT, USA).

#### 4.8.3. Permeability Assay

Co-culture models were prepared by modifying the approach from Costa et al., 2023 [36]. Caco-2 and HT29-MTX cells were co-seeded in a 90:10 ratio in the apical chamber of a 12-well Transwell plate (Corning, New York, NY, USA), forming a monolayer with a density of 1 × 10^5^ cells/cm^2^ per insert. The cultures were maintained for 21 days before analysis. On day 21, the media in the apical chamber was replaced with either plain media (basal control), media with digested samples, or 10% (*v*/*v*) DMSO (stress control), and the plate was incubated for another 6 h. Membrane integrity was evaluated by measuring transepithelial electrical resistance (TEER) with a Millicell ERS-2 Voltohmmeter (Merck, Darmstadt, Germany). Monolayers with TEER values between 150 and 250 Ω·cm^2^ were selected for permeability experiments. The medium-chain fatty acids (MCFAs) were monitored at intervals of 1, 3, and 6 h by collecting 0.5 mL aliquots; the sampled volume was replaced with fresh medium each time. The fatty acid profiles were assessed at these intervals as previously described. All the samples were analyzed in duplicate.

### 4.9. Statistical Analysis

Statistical analysis was performed using GraphPad Prism 10 (Boston, MA, USA). The data were presented as mean ± standard deviation. The normality of the data distribution was confirmed using the Shapiro–Wilk test. The results were analyzed at a 0.05 significance level through one-way ANOVA, followed by Tukey’s multiple comparisons test to determine statistically significant differences between the samples.

## Figures and Tables

**Table 1:** Fatty acid profile of MCFA-loaded bigel and coconut oil.

Fatty Acid	Bigel	Oil
C6	2.13 ± 0.01	42.62 ± 4.54 ****
C8	29.01 ± 0.02	59.84 ± 4.05 ****
C10	21.80 ± 0.09	47.08 ± 3.12 ****
C12	178.32 ± 0.10	412.86 ± 7.85 ****
C14	71.38 ± 0.03	175.91 ± 11.79 ****
C16	42.43 ± 0.02	71.92 ± 4.86 ****
C18	27.88 ± 0.04	29.16 ± 1.86 ****
C18:1 c9	24.56 ± 0.03	45.01 ± 3.18 ****
C18:1 c11	0.46 ± 0.03	0.40 ± 0.05 ^ns^
C18.2	3.19 ± 0.01	7.69 ± 0.53 ****
C18:3	0.65 ± 0.11	0.80 ± 0.09 ^ns^
C22	n.d.	0.18 ± 0.03
∑MCFA	231.25 ± 0.21	893.45 ± 12.0 ****
∑Fatty acids	401.81 ± 0.43	562.39 ± 8.96 ****

Results are expressed in mg/g as mean ± standard deviation of three replicates. C6 caproic acid; C8 caprylic acid; C10 capric acid; C12 lauric acid; C14 myristic acid; C16 palmitic acid; C18 stearic acid; C18:1 t9 elaidic acid; C18:1 t11 vaccenic acid; C18:1 c9 oleic acid; C18:1 c11 cis vaccenic acid; C18:2 c9c12 linoleic acid; C18:3 c9c12c15; C22 behenic acid, MCFA medium-chain fatty acid; n.d. not detected. ns means no significant differences (*p* > 0.05), and **** means significant differences (*p* < 0.001).

## Data Availability

The data presented in this study are available on request from the corresponding author.
